# An inter-disciplinary approach to the energy transition in South Africa

**DOI:** 10.1007/s43621-021-00043-w

**Published:** 2021-07-31

**Authors:** Iain Todd, Darren McCauley

**Affiliations:** 1 Erasmus University Rotterdam, Mandeville Building, Room 17.34, PO Box 1738, 3000 DR Rotterdam, The Netherlands; 2 Chair in the Management of International Social Challenges, Erasmus University Rotterdam, Rotterdam, The Netherlands

**Keywords:** Energy transition, Energy justice, Social inequality, Renewable energy, South Africa

## Abstract

The compelling need to tackle climate change is well-established. It is a challenge which is being faced by all nations. This requires an approach which is truly inter-disciplinary in nature, drawing on the expertise of politicians, social scientists, and technologists. We report how the pace of the energy transition can be influenced significantly by both the operation of societal barriers, and by policy actions aimed at reducing these effects. Using the case study of South Africa, a suite of interviews has been conducted with diverse energy interests, to develop and analyse four key issues pertinent to the energy transition there. We do so primarily through the lens of delivering energy justice to that society. In doing so, we emphasise the need to monitor, model, and modify the dynamic characteristic of the energy transition process and the delivery of energy justice; a static approach which ignores the fluid nature of transition will be insufficient. We conclude that the South African fossil fuel industry is still impeding the development of the country’s renewable resources, and the price of doing so is being met by those living in townships and in rural areas.

## Highlights

- The interdisciplinary nature of an energy transition.
- The implications for four South African energy policies.
- The identification of new research directions for energy justice.

## Introduction

While a nation’s energy infrastructure is always in a process of change, the current energy situation in most countries is particularly fluid, driven by the global moves away from fossil fuels towards renewable energy [1–3]. The need for an energy transition is not only for the purposes of carbon reduction to tackle climate change and improving air quality. It also increases energy security, with nations wishing to meet more of their energy needs from their own resources. The changes are associated with moves from centralised facilities to distributed energy production, and towards cost-effective means of energy storage [4]. These are profound changes which affect not only power generation but also the production of heating and the provision of transport. The energy future will look very different to the present time, and we need energy policy-making that reflects the dynamics of this paradigm shift.

We argue that policy development in the energy sector requires the most acute appreciation of the interplay between the worlds of technology, the social sciences, and politics. Of these, we recognise that while social science is highly influential, it is surprising that the equity and justice implications of energy transitions have only been subject to academic consideration in recent years [5–10]. Energy justice can be understood as a fourth objective to be added to the traditional trilemma of energy objectives—economy, environment, and security. Unlike these three objectives, energy justice gives centre stage to the energy equity aspect of social, economic, and environmental policies [11–13]. It applies within and between past, present, and future generations. Its scope is wide-ranging. Policies must consider how such considerations may be incorporated.

Action is urgently required to tackle climate change. In 2018 the International Panel on Climate Change (IPCC) called for “rapid, far-reaching and unprecedented changes in all aspects of society” to limit global warming to 1.5 degrees C [14]. But progress is slow. According to the BP Statistical Review of World Energy 2020, the share of primary energy produced from renewable sources on a global basis is only 11% [15]. And in South Africa, this figure was a mere 2% in 2019.

This paper addresses the need to move energy transitions onto a truly interdisciplinary basis, encompassing the societal ambitions of the social scientist, the policy development of the political world, and the project management of the business sector. It does so through the case study of the energy transition in South Africa. This requires an awareness of the current energy policy in South Africa, its history, its constraints, how that policy is likely to develop over time, and how it might be adapted further. Swilling et al.assessed the prospects for energy transition in the country [16]. They noted that the South African energy system generates inequalities relating to uneven spatial harms, environmental impacts, and the unequal capacity to mitigate and adapt. They concluded that a just transition is unlikely in South Africa but considered that the falling prices of renewables could reverse that position. Yet, in a more recent document, Vogel and Swilling [17] conclude that South Africa is “ideally positioned to take advantage of the global energy transition”. And in the same document, Mohamed [18] reports the following views of the Council of South African Trades Unions (COSATU):

“A just transition provides the opportunity for deeper transformation that includes the redistribution of power and resources, towards a more just and equitable social order”.

This seems fundamental. According to Guruswamy [19], energy transitions have the potential to alleviate the needs of billions of people who lack access to modern energy provision.

In considering further the need for a more just and equitable social order, this paper invokes the most central and widely supported approach to energy justice—a tripartite structure of tenets comprising distributional justice, recognition-based justice, and procedural justice. This framework has been developed by Fuller and Bulkeley [20] and McCauley et al. [21] and is derived in part from the works of Rawls [22] and Fraser [23]. These tenets have been described by Jenkins et al. [24] using the shorthand of “the What, the Who and the How” of energy justice. They are examined further in the literature review in the Sect. 2 of this paper, which considers also the political and technological factors which are most prominent and most relevant to this issue in South Africa.

Section 3 of the paper describes the methods used to carry out this research. It describes the planning and conduct of a suite of 28 semi-structured interviews with South African energy experts in May–June 2018. These were aimed at identifying the steps necessary to accelerate the energy transition in that country. The answers seemed to lie in the social sciences rather than the technical world, and this is examined further.

The findings of the interviews were gathered pre-COVID and are presented and analysed in Sect. 4 of this paper. This section is structured according to the main components of government energy policy in South Africa. Each is assessed both in terms of the prospects for improved energy justice, seeking to identify any practical steps through which they could be implemented. This is followed by Sect. 5 which discusses the policy implications of the research, advancing how this interdisciplinary challenge would benefit from a greater emphasis on the temporality (dynamics) of energy justice. We submit that such a focus embraces the need to monitor the temporality of the concept, to model its operation with an emphasis on outcome before process, and to modify policies to ensure improved delivery and to tackle policy barriers which may exist. A summary of conclusions is presented in Sect. 6.

## The crossroads of renewable technology, energy social science, and South African energy politics

The case study selected considers the confluence of the technological, societal, and political pressures which are relevant to the energy transition in South Africa. The following sub-sections assess the availability of existing literature in each of these areas.

### Renewable technology

Africa is often referred to as the Sun continent. Parts of Africa experience as much as 4300 h of sunlight a year [25]. According to the World Bank’s Global Solar Atlas [26]—in July 2021—the capital cities of the following sub-Saharan countries would generate annually the following solar electricity per kw of solar cells: South Africa 1800; Botswana 1845; Lesotho 1860; Mozambique 1580; Zimbabwe 1780; Namibia 1975; Angola 1570; Malawi 1700. These are impressive figures in global terms. Further, Africa’s energy demands are set to increase in the next few decades due to major structural transformations in population, urbanisation, and economic growth. The challenge has been appreciated at the highest level, as when President Obama spoke at the University of Cape Town in 2013, to commemorate the centenary of the birth of Nelson Mandela, and to launch the Power Africa initiative [27]:

“Access to electricity is fundamental to opportunity in this age. It is the light that children study by, the energy that allows an idea to be transformed into a real business. It is the lifeline of families to meet their most basic needs, and the connection that is needed to plug Africa into the grid of the global economy.”

This potential has been supported by the South African Photovoltaic Industry Association, pointing out that Africa has 7 of the 10 sunniest countries in the world [28]. This view is reinforced by Gilchrist and Helmund [29] who conducted industry surveys on renewable energy in Africa, concluding that “Renewable energy is the next big thing in Africa—it is going to be the next agent of transformation".

The delivery of that potential to date has however been limited. The UN’s NEPAD initiative has published its assessment of the reasons why renewable energy has been limited in the region of Africa [30]. These researchers considered the potential barriers as: a poor institutional framework and infrastructure; inadequate planning policies for renewables; a lack of co-ordination and linkage in the renewables programme; pricing distortions which have placed renewable energy at a disadvantage; high initial capital costs; weak dissemination strategies; a lack of skilled manpower; poor baseline information; and weak maintenance service and infrastructure.

Brunet et al. [31] have also reviewed the development of solar PV in Africa. They conclude that the most promising role for renewable energies remains with the poorest and most remote populations. However, this opportunity faces several constraints, including cultural aspects, the level of education and training, unstable and weak economies, and inconsistency in energy policies. But in the context of African countries with some of the highest solar irradiance in the world, Brunet considers that PV represents an opportunity to respond to the continent’s industrialisation needs, and other key aspects such as poverty and food security.

The renewable potential of South Africa is best encapsulated by a key study published in 2016 by the South Africa government’s Council for Scientific and Industrial Research (CSIR). This studied the ability of the South African electrical network to incorporate large amounts of solar and wind energy. It reached the following unequivocal conclusions: solar and wind resources in South Africa are of a world-class standard; both have low seasonality; and space is not a constraint to their development. The report’s overall conclusion was that South Africa has the perfect conditions to introduce very large quantities of variable renewables into the electricity system [32]. The installed solar capacity in South Africa is the largest in Africa, and it is expected to rise to 8400 MW by 2030. Most recently, in May 2019, the largest solar farm in South Africa was opened with a capacity of 100 MW. Some progress is being made.

### Energy social science

Energy social science has seen significant research activity in the past decade by a group of researchers applying the principles of energy justice—essentially the concept of fairness—to the global energy transition [8, 21, 33–38]. In doing so they have defined a new genre. McCauley [5] defines the concept as “the application of rights—both social and environmental—at each component part of the energy system”. Jenkins [24] frames the concept as “a new cross-cutting social science research agenda, which seeks to apply justice principles to energy policy, energy production and systems, energy consumption, energy activism, energy security and climate change”. Sovacool and Dworkin [34] define energy justice as “a global energy system that fairly disseminates both the benefits and costs of energy access, and one that has representative and impartial decision-making”. And Heffron [33] provides “an obligation not to diminish the opportunities of future generations to achieve well-being at least equal to those who come before them”. From these publications, the first conclusion is that there is no single definition of energy justice [7, 13, 39]. Different researchers favour different perspectives [40, 41].

Reflecting on the diversity of interpretations, contributors to the study area have proposed terms to describe the constituent parts of energy justice [42–45]. Of these, the most central and widely supported involves a tripartite structure of tenets comprising distributional justice, recognition-based justice, and procedural justice [9, 46, 47].

*Distributional justice* analyses the benefits and burdens associated with energy policy. Guruswami [19] advocates that energy poverty can only be addressed within a framework of distributive justice, as part of the overall right to economic and social development established by the foundational norm of sustainable development. The analysis and interpretation of such a landscape requires an appreciation of many qualitative disciplines, including the geographical, historical, economic, and political aspects of a project.

More studies are becoming available which apply these considerations to real-life major energy projects [48–51]. Such issues can also transcend national boundaries. The widespread movements of energy resources between countries—and through transit countries—confer differing benefits and impose uneven burdens on different societies [42, 52]. The building of large dams such as the Three Gorges in China or the damming of the Nile have major impacts on displaced communities, and influence water supply to agriculture across international boundaries, raising immense issues of energy justice. Such projects emphasise the little-studied time-dependence of distributional justice, building on recent work around temporalities [41].

*Recognition-based justice* moves researchers to examine the above distributional information in terms of one causative aspect—differing degrees of recognition of various sectors of society [11, 42, 45]. According to the UN Forum for Social Development [53], such differences involve specifically the inequalities in the distribution of opportunities for civil and political participation. Walker and Day [54] indicate that such issues can manifest themselves through the choice of location for centralised energy facilities, or policy approaches—overt or unwritten—towards specific sectors of society such as the aged, the disabled, or indigenous communities. Yenneti and Day [55] note large impacts on the livelihoods of rural communities and the further marginalisation of those of lowest status in India. Baker [56] examines the prospects for wind development in Oaxaca, Mexico, presenting a framework for greater participation in renewable energy development by affected communities.

But recognition issues are not restricted to the developing world. Jenkins [57] studies representational concerns at two nuclear facilities in the UK. Healy and Barry [58] advocate that the role of labour in low-carbon transitions must be addressed more systematically. Lacey-Barnacle and Bird [59] examine the critical influence of intermediary organisations, while Hurlbert et al. [60] describe procedural justice innovations in Canada including the constitutional recognition of aboriginal rights and the duty to consult aboriginal peoples. Frameworks of recognition justice must therefore be applicable to multiple contexts in both the developing and developed worlds.

*Procedural justice* has been defined as the study of how decision-makers have sought to engage with communities [61–63]. It is essentially concerned with processes. Weak procedures can explain observed variations in both distributional justice and recognition-based justice [64]. Walker and Day [54] identify three elements which are key to establishing an equitable approach to procedural matters: access to information; meaningful participation; and access to legal processes. Warren and McFadyen [65] demonstrate how fostering a sense of community ownership can enhance the process of acceptance. Other key aspects of the study of procedures include the importance of local knowledge, a study of the flow of information involved in a project, and the representation on decision-making bodies.

Yenneti and Day [55] conclude that the principles of energy justice—providing detailed information, valuing local knowledge, listening to communities through responding to concerns raised—can assist not only the delivery of energy justice but also the acceptance of change by the local community. McCauley et al. [66] point in favourable terms to the consultation efforts of a wind farm developer in Finland. Mundaca et al.[67] have examined two European case studies from the energy justice perspective, citing multiple tensions and conflicts. Roddis et al.[68] analyse the effect that community acceptance has had on planning applications for onshore wind and solar farms in Great Britain between 1990 and 2017. This material clearly demonstrates that real-life projects in a range of countries can fail to deliver fairness through an inattention to equitable procedures.

### The politics of energy in South Africa

This review cannot attempt to encapsulate the full history of energy politics in South Africa, on which subject many papers and books have been written. But to be able to assess the potential for a just transition towards renewable energy, it is necessary to build some understanding of the pressures which have created the status quo, and which are evolving further today.

Before addressing energy issues, mention should be made that South Africa faces severe societal pressures. It has one of the highest social inequality ratios in the world—a Gini Index in 2019 of 0.63 according to Our World in Data [69]. It is noted that a higher Gini Index means greater inequality. The South African figure can be compared to other sub-Saharan countries as follows: Botswana 0.53; Lesotho 0.44; Mozambique 0.54; Zimbabwe 0.50; Namibia 0.59; Angola 0.51; Malawi 0.44. In South Africa, the top 10% of the population earn 58% of the income, while the bottom 50% earn 8% of income. The white population owns 31% of all land in South Africa, including 72% of farming land. In addition, unemployment in the country is in the region of 25% [70]. The currency has seen significant devaluation since 2012 [71]. High rates of domestic crime are a problem. South Africa faces social tensions on a scale not necessarily to be found in all African nations. And there is little doubt—in all societies—that the impact of COVID-19 acts most severely on the poorest of society, so widening inequalities further [72].

Moving to the politics of energy, in its assessment of the prospects for renewable energy, the World Energy Council [73] selected South Africa as one of its case studies. This concluded that South Africa’s main energy challenge is to overcome supply shortages, while diversifying the energy mix. The most important factors considered were providing regulatory clarity to increase investments, replacing fossil fuels with shale gas and renewable energy systems, and improving coordination among government agencies. In electricity, the development of renewable energy capacities will require the construction of additional transmission infrastructure as the current grid connections are primarily fed from large coal plants in the mining areas, while wind and solar resources are in other parts of the country.

## Methods

To conduct a multi-disciplinary assessment of the energy transition in one country, South Africa was selected as the case study location. There were several reasons for this. South Africa is arguably the leading economy in Africa, with huge energy resources both in fossil fuels and renewables potential. It has commenced its journey towards renewable energy, through its Renewable Energy Independent Power Procurement Programme (REIPPP). But compared to the renewable resources available, the progress made to date has been modest. South Africa would therefore seem to perfectly represent the conundrum of failing to exploit readily available renewable energy resources on the African continent. A successful application for travel and accommodation funding was made to the SASIE network—the South African PhD Partnering Network for Inclusive Growth through Social Innovation and Entrepreneurship. This project is jointly funded by the UK’s Economic and Social Research Council (EPSR), the UK Newton Fund, and the South African National Research Foundation.

The principal research question to be addressed in the case study was: How could the energy transition in South Africa be delivered in a way which improves energy justice? This was supported by the more specific questions set out in Table 1 below.

**Table 1:** Key questions

Which societal factors in South Africa relate to an energy transition, notably the increased deployment of solar energy?	
Which key societal barriers are impeding such an energy transition, and what actions could be taken to reduce their effect?	
How could an energy transition deliver improved energy justice?	
How can a transition to improve energy justice be better communicated to a wider audience?	

The principal method of data-gathering selected was to conduct 28 elite semi-structured interviews, with South African energy experts. The use of elite semi-structured interviews provided an approach which was open-ended yet directed, shaped yet emergent, and paced yet unrestricted [74]. They would have a relatively informal style and be based on the premise that knowledge can be produced from such interchanges. The approach was approved by the Ethics Committee of the University of St Andrews.

The 28 interviewees were drawn from public sector officials—both national and municipal, the state electrical utility ESKOM, representatives of industry and trades unions, project developers, university researchers, and NGOs. These are listed in the Appendix. Academic staff at the University of Cape Town provided valuable assistance in setting up the interview schedule, which was conducted in May–June 2018. The establishment of the interview with ESKOM proved most troublesome—including the author being blacklisted by the organisation—but an interview was secured in the end with assistance from others. All interviews were conducted by the lead author, in English, in the cities of Cape Town and Durban. (Admittedly, this interview group had an urban focus; while it would have been of interest and relevance to address the views of the rural sector, the constraints of timing and logistics did not permit this. But we consider that this limitation does not eclipse the core purpose of the research.) All interviews were conducted on a voluntary, confidential, and fully informed basis. The interview schedule included a visit to the Langa township in Cape Town, accompanied by a researcher from the University of Cape Town.

The interview findings were analysed according to the principles of qualitative content analysis, assigning key observations to one of (initially) 13 coding themes. This number was then reduced iteratively to 10 coding themes, following the Grounded Theory Method [75]. This resulted in a balance between sufficient but not excessive differentiation to produce a suitable thematic structure. This process generated 600 key data findings, which were compiled into a Microsoft Excel database. The findings of the interviews are set out in the following Sect. 4.

## Findings

We now describe the interview findings associated with the main interview question—How could the energy transition in South Africa be delivered in a way which improves energy justice? In Sect. 2 we described the technological, societal, and politics pressures which are acting on the energy transition in South Africa. We acknowledge that these are individual contributary elements of a complex societal picture, but in the interview programme—whose methodology is set out in the preceding section—discussion was structured around how these operate in an integrated manner on four key energy policies being implemented by the South African government. These are: the Renewable Energy Independent Power Producer Procurement (REIPPP) project; proposed legislation to restructure the state electrical utility ESKOM; the need to improve the municipal management of townships; and the provision of Free Basic Electricity (FBE) to the poorest sectors of society. These provided a suitable framework within which to assess the prospects for improved energy justice in South Africa, as described in the following sub-sections.

### The renewable energy independent power producer procurement (REIPPP)

To mobilise its energy transition, the South African government instigated its flagship renewable energy policy—the so-called REIPPP project (the Renewable Energy Independent Power Producer Procurement project). This was established on four criteria: local job creation; local ownership; economic development; and socio-economic development. For example, round 4 of the REIPPP requires that 40% of project ownership is black. But it has been noted that poverty alleviation and inequality are not the main drivers of the scheme [76]. By early 2018, the REIPPP project had seen three rounds of implementation, when the government of Jacob Zuma was succeeded by that of Cyril Ramaphosa. One of the first acts of the new administration was to sign the much-delayed contracts for Round 4 of the REIPPP project in April 2018. This was widely welcomed as a brave move by those advocating the energy transition of South Africa’s energy system. These renewable energy contracts are worth 56 billion Rand (£3.5 billion) with independent power producers, and encompass 27 solar and wind projects.

The policy was described in the interview with a member of the REIPPP administration team in the South African government in the following terms:

“The drivers of the REIPPP programme are security of supply, climate change, and affordability, but the objectives do not include social redistribution”.

This was a curious remark, as many other interviewees saw distinct social benefits coming from the policy. The comment might be explained by the government implementing a policy of social restructuring but not wishing to do so overtly. The views of interviewees include those of an academic who had studied the social impact of the REIPPP project):

“Round 4 of the REIPPP requires 40% black ownership of applicant organisations. The socio-economic benefits seem to have worked well, although they have not been audited, nor have they been examined by the academic sector. They also add cost and complexity to projects. Should it be the role of power providers to solve social issues? The REIPPP projects have been effective in terms of engagement with banks and developers.”

This was supplemented by another academic, who advised that job creation under REIPPP is the most important factor for communities. An employee of the state electrical utility ESKOM postulated that:

“Round 5 of the REIPPP project might come forward by the end of 2018. If so, strengthened social conditions might be expected, including increasing the local content requirement to 60–65%.”

On the effectiveness of earlier rounds of REIPPP, an NGO based in Cape Town considered that:

“The REIPPP project has been a big success, but it is not perfect. There is too much control in the hands of private industry, and there has been insufficient public consultation on the scheme.”

A policy officer at the Council of South African Trades Unions (COSATU) assessed the impact of REIPPP in the following terms:

“The community benefit provided by REIPPP and its local content are both insufficient. Also, there are REIPPP projects in off-grid areas where their completion did not result in an adequate electrical supply for the local population.”

A further perspective was provided by a project developer who had worked on REIPPP projects for 6 years:

“The REIPPP schemes I worked on were financed by a South African pension fund (Old Mutual) and by the Nelson Mandela fund. The REIPPP programme is not perfect, but it is working; it is good. Developers go to great efforts to satisfy the social conditions attached to the award of a REIPPP project. These relate to both economic development and social development, and progress against delivery is carefully monitored. Communities need to manage their expectations from the project’s community benefits provisions.”

A second project developer commented as follows:

“The REIPPP programme is a game-changer for the continent. It shows great leadership and sets out the future direction of travel. It is likely to influence neighbouring countries.”

He continued with views on the role of foreign project management, using the phrase “the circus comes and goes”. This displayed perhaps a measure of detachment from the local communities.

These developer views may be supplemented by those of McEwan [76], who noted that community benefit under REIPPP is typically 1–1.5% of the project income. Tait [77] considers that there are four REIPPP criteria to be satisfied—local job creation, local ownership, economic development, and socio-economic development. Areas of concern noted include engagement with local communities, the issue of defining the boundaries (50 km) of community benefit, and how to manage circumstances where nearby projects have overlapping community benefit zones. Similarly, Davies et al. [78] propose that REIPPP projects should have enhanced social impacts, should be better integrated with the local economy, and that there should be greater coordination between REIPPP projects. They pose an interesting question—whether REIPPP projects should have to pay local taxes and rates to local authorities, in addition to community benefits. The authors note this is exactly the policy approach which has been developed in the UK in recent years, with a resultant negative impact on the growth of solar and wind projects.

In terms of energy justice, this policy clearly impinges on the delivery of distributive justice. The REIPPP projects are clearly aimed at the reduction of social inequality in South Africa, although there are inevitable comments that the degree of support is not sufficient. It is less focussed on recognition-based justice. But procedural justice is once again key—the views of academics, project developers, NGOs and the public sector agree on this point. The processes involved must be viewed as equitable to secure the support of societal groups. Overall, this policy can be judged to be delivering improved energy justice.

### Proposed legislation to restructure the state electrical utility ESKOM

The South African state electrical utility ESKOM occupies a central position in terms of the country’s national energy strategy. It is the largest generator of electricity in Africa [79] and is described by Jaglin and Dubresson [80] as the 6th largest company in Africa. ESKOM’s comprehensive suite of responsibilities and powers include power generation, transmission, and distribution. In this way it parallels the monopoly position of the UK’s state utility—the CEGB—prior to its privatisation in the 1980s. That process was known as unbundling, and a similar process has been under discussion in South Africa for some time. Legislation has been prepared to do so—the so-called ISMO Act (Independent System Market Operator)—but it has never been implemented. In the words of one NGO:

“The debate is between those in favour of unbundling and old-style leftist control. The main barrier is the need to unbundle the electricity sector—i.e., the state utility ESKOM—and so transform the electricity market.”

Another NGO put it even more simply, “ESKOM needs to be reformed”, even if they are “resisting … partial unbundling” as commented one Government employee. One academic noted that “ESKOM is not built for change”. A trades union representative considered that “ESKOM acts independently of the Energy Minister, and there is a need for cultural realignment” especially considering their “immense political influence” according to one academic. An NGO added: “Such are the problems facing the utility, the collapse of ESKOM is not impossible, nor the chance of energy prices going sky-high”. One interviewee from the Municipality of Cape Town referred to a “utility death spiral”, to describe a utility facing continually falling income and rising costs.

However, it is also important to remember that utility restructuring—while bringing cost savings—can carry a risk of a loss of strategic direction. This can manifest itself as a lack of clarity on the responsibility to “keep the lights on”, and also providing long-term strategic investment in energy research. Both of these observations were noted in the UK example.

### Improvements to the municipal management of townships

A third policy area relates to the municipal management of townships in the major cities of South Africa, where energy injustice is perhaps most apparent. In June 2018, the author visited the Cape Town township of Langa in the company of a researcher from the University of Cape Town, who explained:

“The township has a population of 50,000 and covers an area of 3 square kilometres. Its name means the Sun, in Khosa. It was the first black township—established in 1927—and over its history it has acted as a centre of resistance to apartheid. Several inhabitants were killed in 1980. In 2005, a cultural centre was built. In 2014 there were further violent protests, with shops looted and burned. In 2016, 55% of the murders in Cape Town occurred in this township—there is a culture of gangs in control of the township. There are serious social issues—unemployment rates in the township are over 30%.”

One of the NGOs further noted:

“Some township residents spend 20% of their income on energy. And that most non-township people in South Africa had never set foot in a township.”

In terms of energy provision, the township had street lighting, and satellite dishes were ubiquitous. Satellite television is viewed as a status symbol; solar heating systems had also been installed in some parts of the township.

Another NGO described the planned investment in the Atlantis township project, which is also in Cape Town, at an existing off-grid rural settlement. The municipality is an active participant, as is the regional government, who had designated the project as a Special Economic Zone (SEZ). They described how this community of 100,000 had a gangland reputation, but they were seeking to build community involvement, through the active participation of churches and schools. They had assembled a “dream team of social, technical and financial service providers”, and had opened an office in the township. A key requirement was the need to recruit anchor tenants and establish a role for local businesses. They noted that “money is not an issue—there is plenty of finance available for suitable projects”. But social issues are very prevalent. They advised that plans included solar-powered street lighting “to avoid women and children being raped on their way to the toilet area”. Comments relating to the risk of violence in communities arose in several interviews.

A further NGO described the energy provision in the townships as “second-class”. They suggested that townships could consider leasing roof-top space for solar power, although remarked that there would be a risk of the panels being stolen. Another NGO advised that there are significant areas of townships which refuse to pay for electricity, or which benefit from illegal connections. There are further complications over “backyarders”, whereby township residents share an electrical connection between several family units on one property/meter.

### Free basic electricity (FBE)

In 2003, the government of South Africa introduced its policy of Free Basic Electricity. It is a policy which is radical and socially redistributive. In the words of one NGO:

“Free Basic Electricity (FBE) is a government policy by which the poorest in society receive 100 kwh of free electricity per month, and the next 100 kwh is subsidised. This cross-subsidy provides valuable and widely supported help to the poor. The policy is radical, socially re-distributive, and it is working well; it is an industrial contribution to social alignment. However, the rural poor could be disadvantaged by any future concentration on the needs of an increasing urban population.”

Another NGO advised that poorer families still benefit from the Free Basic Electricity (FBE), but ESKOM struggled to keep up with the work demand. An academic based in Cape Town advised that:

“The Free Basic Electricity policy seems to have worked very well. But there has not been any auditing of the policy; academics are not studying it. The policy adds costs to projects, adds complexity, and one can question whether power projects should be funding social change.”

Officials at the Municipality of Cape Town advised that the risk of weakening such redistributive advantages is very real. An NGO noted that in informal settlements the poor can run out of their monthly FBE after a couple of weeks and had to revert to using wood or paraffin. This coincides with the published findings of Mzolo [81], who reports that mixed-fuel provision is the daily practice for the poorest sectors of South African society.

In terms of energy justice, this policy is clearly focussed on the delivery of distributive justice. It impinges on recognition-based justice—supporting the energy needs of disenfranchised groups who might otherwise not be considered by government. It is also relevant to procedural justice—interviewees reported that the machinery seemed to work well, although noting some areas which could be improved—the adequacy of provision, the qualifying threshold for the benefit, and the complexity of “backyarder issue” prevalent in township areas. But overall, the policy is an excellent example of the delivery of energy justice. It was widely admired in the interviews held, and it was no surprise to learn that some other countries—such as India—are considering a similar policy.

## Discussion

We have described in Sect. 2 the current position of South African energy policy through a literature review, and in Sect. 4 the findings of a suite of interviews conducted in South Africa during 2018. We now assess the implications of these findings on the energy transition in South Africa from the three original multi-disciplinary perspectives of technology, social science, and politics. This section is followed by our overall conclusions in Sect. 6.

### Technological considerations

The literature review and the interviews agree that any barriers to the energy transition in South Africa do not lie in the realm of technology. The conclusions of the CSIR report of 2016—reported in Sect. 2—are key to this finding. No interview raised technological barriers as an explanation of the limited take-up of renewable energy in South Africa. This finding can also be interpreted as applicable to many African nations—the technologies of both solar and wind power are readily available, applicable, and becoming ever more affordable. They are modular and so scale-able, and highly suited to distributed energy applications. Their application is considerably assisted by the concomitant growth of energy storage technologies.

This conclusion is therefore not a surprise, as globally the energy transition is accelerating the growth of renewable energy, delivering innovation, and providing economies of scale. We consider that the reasons for the limited take-up of renewable energy in South Africa must lie in the non-technological areas.

### Social science

We have considered the complexity of societal pressures in South Africa in both the literature review and in the interviews. Again, there is significant agreement. If anything, the interviews have demonstrated an even more polarised view between the proponents and opponents of the energy transition in South Africa. This is characterised by a recourse to legal action by both sides and by a lack of progress on dealing with the challenges of township deprivation. As described in the literature review, it is possible to structure a discussion of the fairness of this situation in terms of distributive justice, recognition-based justice, and procedural justice.

Distributive justice relates to the fairness of allocating the benefits and burdens of energy provision among the different sectors of society. This is a very political objective, fraught with the potential for differing opinions and different perceptions of priority, effectiveness, and fairness. Any major change inevitably leads to winners and losers, and the task of government must be to produce an outcome which maximises the benefits for the most people. There are however significant challenges: political influence may be unfairly divided, e.g., between rural and urban, or between developed and developing nations; economic systems may favour some elements of society over others; institutions may be constituted to favour incumbent energy providers; government policy may not deliver a just distribution of energy or energy access.

So, do the interviews indicate an equitable outcome on the distribution of energy? While providing apparently clear commitments to an energy transition, there was much evidence that energy distribution is powerfully influenced by both socio-cultural issues and the incumbent power of the mining industry/workforce. A recent publication has termed the latter influence as “incumbent resistance” [82]. Such influence is referred to as “critical” by Heiskanen et al. [83], in view of the financial strength of the incumbents, and their control of the existing energy infrastructure. The interviews also reinforced the temporal nature of energy justice, evolving over time, making steady progress with occasional setbacks, as relevant policies come and go.

The interviews and literature review have therefore reinforced the important role of distributional aspects in energy justice. One shining light is the redistributive policy of Free Basic Electricity; this is under consideration by several other countries in the world and here South Africa does seem to be ahead of the field.

Recognition-based justice relates to the equality of voice in the process of an energy transition. The risks posed by inappropriate representation include: a lack of consideration provided to all sectors, such as disadvantaged members of society; industry giving inadequate recognition to all parties affected by renewable projects; and institutions not recognising all affected parties. This may be traced back to inadequate policies and regulations which do not give enough say to all in society. Or insufficient employment or training opportunities leading to the aspirations of all sectors not being represented.

Fraser [84] advances that the struggle for recognition is the key form of political conflict, supporting the concept of recognition of difference as a necessary requirement of achieving justice. Examples given include nationality, ethnicity, race, gender, and sexuality. All such thinking finds ready application in modern South African society, which notably agrees with the observation by Madumo [85] that South Africa’s Gini Index has increased dramatically since the end of apartheid in 1994. The need for tracking energy justice over time is therefore reinforced here also. Fraser concludes by recommending that the concept of recognition should recognise and defend such cultural politics of difference, in combination with the social politics of equality.

In the interview programme, several comments addressed the need for recognition. The government’s commitment to consultation was considered a significant improvement over the practices of the previous regime. The trade union COSATU considered that off-grid communities—principally in rural areas—are under-represented. They cited examples where off-grid communities were adjacent to power lines taking renewable energy from their vicinity only to meet the needs of urban dwellers, or the mining industry.

The combined effect of interviews and literature review on recognitional aspects of energy justice in South Africa has supported the importance of this objective. Some deficiencies in representation have been noted, but also some signs of improvement in addressing the voices of poorer elements of society. There is an inevitable linkage to the many broader societal pressures facing South Africa, not all of which can be resolved solely through an energy transition, even a successful one.

Procedural justice requires that the processes of change be managed in a way that is fair, not only in representation, but through a conduct which is transparent, logical, balanced, and based on factual evidence. Baker [86] notes that if decarbonisation is to achieve a just transition, then it must also address questions of economic inequality and welfare, referring to the poor having largely been excluded from policy processes in South African society.

The risks of failing to deliver this objective are numerous: politicians may be driven by short-term pressures to act in a non-procedural manner; public sector procedures may be weak or may not be followed with rigour; project procedures may be inadequate—driven by commercial pressures—in areas such as the provision of information; and the planning process may under-estimate local issues and concerns. A focus on procedural aspects—almost by definition—implies a study of how concepts vary over time, and how such variations may be monitored, interpreted, and modified where necessary.

Two examples of governmental procedures aimed at delivering improved energy justice emerged in the interviews. The first is the legislation which requires a proportion of project organisations to be of local content—for example, the REIPPP projects are required to have 40% local content. This is linked to key legislation—the Black Economic Empowerment (BEE) laws—which date from 2003 and which are aimed at redressing the inequalities of apartheid. The second legislative policy relates to the provision of Free Basic Electricity (FBE) to the poorest elements in society, as discussed above. This policy received widespread support among interviewees, who spoke with pride of its redistributive success.

However, the authors consider that several research directions are worthy of further development. These may be grouped under the concept of the dynamics of energy justice, a subject which has received little research attention. Energy justice is characterised by fluidity, and this is particularly relevant when circumstances are changing, as in an energy transition. The associated insights can help to predict how an energy system will evolve into the future.

### Political

The REIPPP decision was closely followed by the publication—in August 2018—of the government’s draft Integrated Resource Plan 2018 [87]. This is the latest in a series of such plans published at intervals of several years—for example, past documents had been published in 2010 and in 2016. The 2016 document was the subject of much criticism by those advocating new forms of energy generation, and the 2018 document was widely regarded as a significant improvement. Since the publication of the draft plan, the government has held hearings before a Parliamentary Committee, taking further evidence on the subject, and this resulted in the publication of the final version of the document in 2019. However, by December 2019, the rolling blackouts in the country involved the loss of 6000 MW of capacity—of which 2000 MW was reportedly “due to sabotage, causing devastating effects on the economy” [88, 89]. The difficulties of energy politics continue to have a major impact on all aspects of South African life.

Our findings on ESKOM complement similar observations in existing research. Rossouw [90] describes “corporate governance breaches of the worst kind”, “exorbitant remuneration”, and “ESKOM serving as a conduit to transfer government resources to corrupt individuals and families”. These observations mirror the views of Hofstatter [91], that “the plunder of ESKOM … brought the economy to the brink of collapse”. And those of Baker and Sovacool [71], who portray ESKOM as a “coal-fired, crisis-ridden, state-owned monopoly electricity utility”.

The acid question is therefore whether a major restructuring of the state utility would improve the prospects for energy justice in South Africa. The interviews and published literature are unanimous that such change would benefit the country. The President is keen to do so, as announced at Davos in January 2019, and it was separately announced [92] that the process would be assisted by Chinese finance. However, this approach generated substantial resistance from the combined incumbent community of ESKOM, the mining industry, and the mining union (NUM), which are sometimes referred to collectively as the Energy-Minerals Complex. Subsequent developments during 2019 involved: the threat of a national strike by COSATU; the NUM criticising the unbundling of ESKOM as the first step on the road to privatisation of the utility [93]; and ESKOM issuing a statement that such industrial action would risk needing to escalate its programme of load-shedding. The government withdrew its plans to restructure the ailing organisation. The authors conclude that a major restructuring of ESKOM would indeed be beneficial.

## Conclusions

Our research has examined the prospects for energy transition in South Africa, and has revealed a complex interplay between the technological, societal, and political pressures acting on that subject. It is surprising that the South African fossil fuel industry—allied with the state electrical utility ESKOM—is still exerting such a blocking influence on the country exploiting more fully its immense renewable resources. The societal elements which are paying the price for this resistance are those living in townships and rural areas. There can be no doubt that the people who live in the township areas of major South African cities are prime candidates for improved energy justice. They deserve the benefits of an equitable distribution of energy benefits, meaningful recognition, and processes which are genuinely consultative in nature. Their needs are not of course limited to energy services, but also education, health, employment prospects, water supply and sanitation, and infrastructure such as roads. But the interviews confirmed that in terms of energy services—which were described at best as second-class—there remains much to be done to achieve energy justice.

We would also conclude that the traditional approach to energy justice places too heavy an emphasis on processes rather than outcomes. We argue that energy justice must be assessed in the context of the outcomes of energy transition, and those outcomes include not only energy fairness but also decarbonisation, energy security and energy affordability. Of these energy objectives, it would seem self-evident that decarbonisation is the principal outcome, without which the concept of an energy transition lacks any meaning. Linking energy justice more closely to the global decarbonisation movement would also have the effect of increasing its understanding, its profile, and its prospects for success. While the balance between outcomes and process has been little addressed in the literature, one exception [94] addresses exactly this point. By considering the balance between process and outcome, they advance that the proactive management of transitions has the potential to help secure improved energy justice.

We note also that the pace of implementation of an energy transition can be influenced significantly by both the operation of societal barriers seeking to impede a transition, and by policy actions aimed at redressing such effects. This aspect is especially important, as it is not only critical and rate-determining, but it is also highly under-researched and under-appreciated. We recommend that the academic field of energy justice be extended further to address the time-variation (or dynamics) of energy justice, on a multi-disciplinary basis.

As for the South African government, we recommend firstly that they must remain committed to a transition to renewable energy and accelerate its implementation wherever possible. To do so, it is essential that it adjusts its policy proposals to the goal of building a powerful coalition of constituents in favour of the energy transition. Our discussion of policy barriers has highlighted some of building blocks needed for such a coalition. The government needs to attract more foreign investment for large-scale renewable energy projects, which has proved highly successful in the REIPPP project to date. Action to restructure ESKOM is also crucial, in order to transform a major obstacle into a force for change, despite political pressures from mining and labour constituencies not to do so. Its financial difficulties could be resolved by selling it off to foreign companies that would be committed to the energy transition. This would result in an increased involvement of overseas expertise and finance to bring in new energy systems. The resources from the sale could also contribute to building political support for the energy transition in the country—including a more active role for South Africa’s major municipalities who could become a powerful constituency in favour of the energy transition.

## Data Availability

The data used can be made available upon request to the corresponding author.

## Appendix

### List of organisations interviewed

1. Independent project developer
2. Energy consultant, formerly University of Cape Town
3. Project developer
4. Green Cape
5. University of London
6. Municipality of Durban
7. University of Cape Town
8. Sustainable Energy Africa
9. Government REIPPP team
10. University of Cape Town Business School
11. Centre for Environmental Rights
12. Project 90 by 2030
13. Municipality of Cape Town (1)
14. Municipality of Cape Town (2)
15. Forethought Africa
16. University of Cape Town
17. ESKOM
18. Consumer
19. Department of Science and Technology
20. Shell
21. Council of South African Trades Unions
22. Scotland Africa Business exchange
23. Mainstream
24. Scottish Renewables
25. Green Cape (re-interview)
26. Centre for Environmental Rights (re-interview)
27. Project 90 by 2030 (re-interview)
28. Sustainable Energy Africa (re-interview).

## Code availability

The only code used for this work was Microsoft Office.
